# Stem Cell and Cell‐Free Strategies for Osteoarthritis: Toward Durable Regenerative Therapies

**DOI:** 10.1155/sci/6699734

**Published:** 2026-06-05

**Authors:** Andreas Pambis, Tianle Li, You Li, Biao Li, Christopher Kim

**Affiliations:** ^1^ Schroeder Arthritis Institute, Krembil Research Institute, University Health Network, Toronto, Ontario, Canada, uhn.ca; ^2^ Department of Basic Medical Science, Faculty of Science and the Schulich School of Medicine and Dentistry, Western University, London, Ontario, Canada, uwo.ca; ^3^ Department of Internal Medicine, Cleveland Clinic Florida, Weston, Florida, USA, clevelandclinic.org

**Keywords:** cartilage, chondrocyte, mesenchymal stromal/stem cells, osteoarthritis, stem cell-free derivatives, synovitis

## Abstract

Osteoarthritis (OA) is the most prevalent degenerative joint disease and a leading cause of pain and disability, yet current treatments remain largely palliative and fail to alter disease progression. Stem cell‐based therapies offer a promising alternative, with mesenchymal stromal/stem cells (MSCs) representing the most clinically advanced approach. Preclinical and clinical studies demonstrate that MSCs exert anti‐inflammatory, immunomodulatory, chondroprotective, and analgesic effects primarily through paracrine signaling rather than durable engraftment. Clinical trials consistently confirm intra‐articular (IA) MSC safety and symptomatic benefit, though structural outcomes remain variable owing to heterogeneity in cell source, dose, and trial design. This recognition has fueled interest in cell‐free derivatives, particularly extracellular vesicles (EVs), which recapitulate MSC paracrine functions while offering improved safety, scalability, and regulatory compatibility. In parallel, bioengineering innovations, including hydrogels, scaffolds, 3D bioprinting, nanotechnology, and genetic enhancement, are being leveraged to prolong persistence, optimize delivery, and enable adaptive, multimodal repair. Emerging sources such as induced pluripotent stem cells (iPSCs), embryonic stem cells (ESCs), and joint‐resident progenitors further expand the regenerative toolkit. These developments suggest that future regenerative strategies for OA may combine cellular therapies, cell‐free products, and biomaterial delivery systems to improve treatment durability and therapeutic control.

## 1. Introduction

Osteoarthritis (OA) is the most common degenerative joint disease and a major cause of chronic pain and disability worldwide [1]. It is characterized by progressive cartilage loss, synovial inflammation, subchondral bone remodeling, and persistent pain, leading to impaired mobility, reduced quality of life, and a substantial socioeconomic burden [2, 3]. Increasing evidence suggests that OA is a whole‐joint disease involving complex interactions among cartilage, synovium, subchondral bone, and inflammatory mediators. However, current treatment options, including analgesics, intra‐articular (IA) corticosteroids, hyaluronic acid (HA) injections, and ultimately joint replacement, remain largely palliative, providing short‐term symptom relief without addressing the underlying joint pathology [4, 5].

In response to these limitations, regenerative medicine has emerged as a promising paradigm, shifting the focus from symptom management to potential disease modification. Among the various stem cell‐based interventions, mesenchymal stromal/stem cells (MSCs) are the most extensively investigated. MSCs can be derived from bone marrow, adipose tissue, synovial tissue, umbilical cord, and placenta and have been evaluated in both preclinical and clinical studies. While alternative sources such as pluripotent stem cell‐derived chondrocytes and joint‐resident progenitors remain at earlier stages of development, MSCs currently represent the most widely studied cell‐based therapy for OA.

Early studies focused on the potential of MSCs to directly regenerate cartilage. However, accumulating evidence indicates that their therapeutic effects largely arise from paracrine signaling, including the secretion of growth factors, extracellular vesicles (EVs), and immunomodulatory mediators that influence the joint microenvironment [6, 7]. This conceptual shift has broadened interest toward MSC‐derived cell‐free derivatives, particularly EVs, which may offer advantages in safety, scalability, and regulatory compatibility. In parallel, advances in bioengineering, including hydrogels, 3D‐bioprinted scaffolds, and synthetic biology tools, are being explored to enhance MSC retention, augment therapeutic potency, and enable controlled therapeutic responsiveness [8, 9].

Despite encouraging progress, translation into routine clinical use remains incomplete. Clinical trials consistently demonstrate that IA MSC administration is safe and improves pain and function; however, evidence for durable structural cartilage repair remains limited and inconsistent [10–12]. Addressing challenges such as product heterogeneity, optimal dosing, delivery strategies, and biomarker integration will be essential to realize the full therapeutic potential of stem cell‐based interventions in OA.

Several recent reviews have summarized MSC‐based therapies and MSC‐derived EVs for OA [13, 14]. However, these studies often address cell‐based and cell‐free approaches separately. In this review, we examine the biological mechanisms underlying MSC therapy, evaluate current clinical evidence, and discuss advances in MSC‐derived EVs, bioengineering strategies, and emerging stem cell sources. By integrating these perspectives, we aim to highlight both the therapeutic potential and the remaining translational challenges that should be addressed to achieve disease‐modifying regenerative therapies for OA.

## 2. Biological Mechanism Underlying MSC Therapy in OA

**Figure 1:**
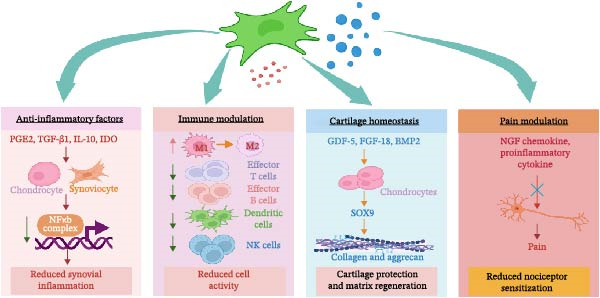
Mechanisms of MSC‐mediated immunomodulation in OA. MSCs regulate inflammation, immune responses, cartilage homeostasis, and pain signaling in OA through paracrine mechanisms. The green cells represent MSCs; blue particles indicate EVs; red dots denote bioactive mediators. Created with BioRender.com.

Current understanding suggests that MSCs influence OA progression mainly through their secretory activity and immune regulation rather than through long‐term engraftment or direct differentiation within the joint [6]. Importantly, these mechanisms are not independent processes. Inflammation, immune activation, cartilage catabolism, and pain signaling interact closely within the OA joint environment, meaning that modulation of one pathway often influences several others (Figure 1).

### 2.1. Anti‐Inflammatory and Immunomodulatory Effects

OA joints are characterized by elevated levels of pro‐inflammatory cytokines such as IL‐1β, TNF‐α, and IL‐6, which activate NF‐κB signaling and promote cartilage matrix degradation through increased expression of catabolic enzymes, including MMP‐13 and ADAMTS‐5 [15]. Persistent inflammatory signaling also disrupts cartilage homeostasis and contributes to progressive tissue degeneration.

MSCs counter this inflammatory microenvironment primarily through paracrine and immunomodulatory mechanisms. They secrete anti‐inflammatory mediators such as prostaglandin E2 [16], TGF‐β1 [17], and indoleamine 2,3‐dioxygenase (IDO) [18], and under inflammatory stimulation can produce additional immunoregulatory cytokines, including IL‐10 [19]. These factors suppress NF‐κB activation in chondrocytes and synoviocytes, reduce the production of inflammatory mediators, and help restore joint homeostasis.

In addition to directly attenuating inflammatory signaling, MSCs regulate immune cell activity within the synovial microenvironment. MSCs promote macrophage polarization from pro‐inflammatory M1 phenotype toward anti‐inflammatory M2 phenotypes [20] and enhance regulatory T cell (Treg) response and inhibit activation of effector T cells, B cells, dendritic cells, and NK cells [21]. Through this coordinated immune modulation, MSCs establish a local environment that limits inflammation and supports cartilage preservation and repair. Consistent with these mechanisms, IA administration of MSCs in rodent OA models reduces synovial IL‐1β and TNF‐α levels and improves histological cartilage integrity [22]. Similarly, bone marrow‐derived MSC (BM‐MSC) exosomes suppress IL‐1β‐induced expression of MMP‐13 and ADAMTS‐5 [15], illustrating how MSC‐derived paracrine factors can modulate inflammatory signaling and preserve cartilage homeostasis.

### 2.2. Cartilage Regeneration and Chondroprotection

MSCs indirectly support cartilage repair through trophic factors that promote chondrocyte survival and anabolic activity. Secreted proteins such as GDF‐5, FGF‐18, and BMP‐2 stimulate SOX9 expression and the synthesis of aggrecan and type II collagen [23]. In preclinical OA models, MSC administration reduces cartilage erosion and enhances proteoglycan deposition [24]. EVs contribute as well; for example, exosomes enriched in miR‐140‐5p enhanced cartilage matrix synthesis and delayed OA progression in rats [25]

### 2.3. Pain Modulation

Pain, the dominant symptom of OA, is modulated by MSCs through both anti‐inflammatory and neuroimmune pathways. By damping inflammatory mediators such as NGF and key chemokines and cytokines [26], MSCs indirectly reduce nociception. In animal models, MSCs improved gait and weight‐bearing [27, 28], while their EVs reduced glial activation and neuroinflammation [29]. Additional mechanisms include prostaglandin E2‐mediated suppression of joint inflammation [30] and exosome‐driven inhibition of subchondral angiogenesis [31]. Clinical trials further support these mechanisms, with IA BM‐MSCs injection reducing synovial inflammation and improving pain and function [32].

Generally, MSCs exert coordinated effects on inflammation, immune regulation, cartilage homeostasis, and pain signaling. By targeting multiple pathological processes involved in OA, these mechanisms provide a strong biological rationale for the clinical translation of MSC‐based therapies, which is discussed in the following section.

## 3. Clinical Translation of MSC Therapies in OA

The clinical translation of MSC therapy for OA has advanced stepwise, beginning with early‐phase trials that established the safety and feasibility. Across open‐label and small phase I/II cohorts, IA MSC administration was consistently well tolerated, with only mild and transient local adverse events. Many studies also reported symptomatic improvement with occasional MRI evidence of structural stabilization (e.g., autologous adipose‐derived MSCs [AD‐MSCs] and BM‐MSCs, stromal vascular fraction, and placental‐derived products) [32–35]. To capture this evolving landscape, we compiled Table 1 summarizing representative clinical trials. Building on these findings, randomized controlled trials (RCTs) rigorously assessed efficacy across diverse MSC sources, including adipose tissue, bone marrow, and umbilical cord, against placebo or active comparators such as HA. These RCTs employed standardized outcomes (WOMAC, KOOS, and VAS) and MRI assessments, tested varied doses and delivery regimens, enrolled broader patient populations, and extended follow‐up to examine durability of response [12, 36–50]. In short, these data underscore the maturation of MSC translation in OA.

**Table 1:** Clinical trials of MSC‐based therapies for OA in this review.

Authors	Registration number	Study design	Patient number	MSC source	Delivery route	Dose	Control group	Endpoints	Key findings
Jo et al. [10]	N/R	Phase I dose‐escalation (proof of concept)	18 (low *n* = 3; mid *n* = 3; high *n* = 12)	Autologous AD‐MSCs	Intra‐articular	10 × 10^6^, 50 × 10^6^, or 100 × 10^6^ cells	None	WOMAC, KOOS, KSS, MRI, safety	Dose‐dependent improvement; best with high‐dose; no SAEs
Kim et al. [11]	NCT03990805	Phase III, multicenter RCT, double‐blind, placebo	89 (AD‐MSC *n* = 46; placebo *n* = 43)	Autologous AD‐MSCs	Intra‐articular	Single IA (dose per protocol)	Placebo	VAS (walking pain), WOMAC, MRI, safety	Significant pain reduction at 6 months; favorable safety profile
Vega et al. [12]	NCT01586312	RCT, double‐blind, HA‐controlled	30 (BM‐MSC *n* = 15; HA *n* = 15)	Allogeneic BM‐MSCs	Intra‐articular	Single IA: 40 × 10^6^	HA	WOMAC, VAS, MRI T2, safety (12 months)	Improved pain/function, MRI vs. HA; no safety issues
Chahal et al. [32]	NCT02351011	Phase I/IIa open‐label	12	Autologous BM‐MSCs	Intra‐articular	1, 10, or 50 × 10^6^ cells	None	KOOS, WOMAC, MRI, biomarkers, safety	PROM improvements; dose‐dependent effects; reduced synovial inflammation; no SAEs; minor AEs in some patients
Emadedin et al. [33]	NCT01504464	Randomized, triple‐blind, placebo‐controlled	43 (19 MSC; 24 placebo)	Autologous BM‐MSCs	Intra‐articular	Single IA: 40 × 10^6^ cells in 4 mL	Placebo (saline)	VAS, WOMAC, walking distance, flexion, safety at 3 and 6 months	Significant improvements in pain/function; no serious AEs
Fodor and Paulseth [34]	NCT02726945	Prospective open‐label feasibility study	Not 6 patients (8 knees)	Autologous adipose‐derived stromal cells (ADSCs)	Intra‐articular	14.1 ± 2.6 × 10^6^ cells per knee	None	KOOS, physical function, AEs	Improved KOOS/physical function; only mild swelling
Soltani et al. [35]	IRCT2015101823298N	Double‐blind, placebo‐controlled pilot RCT	20 (10 MSC; 10 saline)	Allogeneic placental MSCs	Intra‐articular	0.5–0.6 × 10^8^ cells in 10 mL	Saline	VAS, KOOS, ROM, MRA, safety at 24 weeks	Early pain/ROM gains; 10% chondral thickness increase; no serious AEs
Shapiro et al. [36]	NCT01931007	RCT, double‐blind, placebo‐controlled	25 (BMAC *n* = 15; saline *n* = 10)	Bone marrow aspirate concentrate	Intra‐articular	Single injection (per protocol)	Placebo	Pain/function over 12 months, safety	No difference vs. placebo; both improved
Kuah et al. [37]	ACTRN12615000258550	RCT, double‐blind, placebo‐controlled dose‐finding	20 (low‐dose *n* = 10; high‐dose *n* = 10)	Allogeneic human AD‐MSCs (Progenza)	Intra‐articular	Single IA; low vs. high dose	Placebo	Safety, NRS pain/function, biomarkers	Good safety; pain reduction signals
Lee et al. [38]	NCT02658344	Phase IIb RCT, double‐blind, placebo‐controlled	24–30 (per article; randomized to high/low dose vs. placebo)	Autologous AD‐MSCs	Intra‐articular	Low vs. high dose (per protocol)	Placebo	Pain/function, MRI, safety	Clinical improvement vs. placebo; safe
Lamo‐Espinosa et al. [39]	NCT02123368	Long‐term follow‐up of RCT (4 years)	30 (follow‐up)	Autologous BM‐MSCs	Intra‐articular	10 × 10^6^ or 100 × 10^6^ (as per randomization)	HA	WOMAC, VAS, MRI over long term	Sustained benefit in high‐dose group; no major safety issues
Matas et al. [40]	NCT02580695	RCT, double‐blind, HA‐controlled	29 (single *n* = 8; repeated *n* = 9; HA *n* = 12)	Allogeneic UC‐MSCs	Intra‐articular	20 × 10^6^ cells; single vs. repeated IA	HA	WOMAC, VAS, MRI, safety (12 months)	Repeated dosing improved WOMAC more than HA; safe
Chen et al. [41]	NCT02784964	Phase I/II RCT, patient‐blinded, active‐controlled	57 randomized (43 ADSC; 14 HA)	Allogeneic AD‐MSCs (ELIXCYTE)	Intra‐articular	Single IA: 16 × 10^6^, 32 × 10^6^, or 64 × 10^6^ cells	HA	Primary: WOMAC pain; Secondary: WOMAC, KOOS, EQ‐5D, SF‐12, AEs	ADSC groups had earlier and sustained improvements vs. HA; no serious AEs
Freitag et al. [42]	ACTRN12615000258550	Randomized, double‐blind, placebo‐controlled	30 randomized (3 groups)	Allogeneic AD‐MSCs	Intra‐articular	Single vs. two IA injections (100 × 10^6^)	Placebo (HA) Injection	WOMAC, QoL, safety up to 12 months	Safe; trends to clinical improvement vs. placebo
Garza et al. [43]	NCT02726945	Randomized, double‐blind, placebo‐controlled	39 (SVF high‐dose, SVF low‐dose, placebo)	Autologous stromal vascular fraction (SVF)	Intra‐articular	Low vs. high‐dose SVF (TNCs, per paper)	Placebo (saline)	WOMAC (pain, stiffness, function), safety	SVF improved WOMAC pain; no significant safety issues
Ho et al. [44]	CUHK_CCT00469	Randomized, double‐blind, HA‐controlled	20 (BM‐MSC *n* = 10; HA *n* = 10)	Autologous BM‐MSCs	Intra‐articular	6 mL, 1 × 10^6^ cells/mL (single)	6 mL Synvisc‐One (HA)	KOOS, VAS, MRI, safety	BM‐MSCs improved symptoms vs. HA; good safety
Lu et al. [45]	NCT02162693	Phase IIb RCT, double‐blind	53 randomized	Autologous adipose‐derived haMPCs	Intra‐articular	Single IA injection, 50 × 10^6^ cells	HA	WOMAC, VAS, SF‐36, MRI (cartilage), safety	≥50% WOMAC improvement favored re‐join; increased cartilage volume; VAS, QoL improved; no serious AEs
Sadri et al. [46]	IRCT20080728001031N23	Phase II, triple‐blinded, placebo controlled, randomized trial	*N* = 40 (20 MSC, 20 control)	Allogeneic AD‐MSCs	Intra‐articular	IA injection	Placebo	Cartilage regeneration, inflammation modulation, pain	Allogeneic adipose MSCs improved cartilage regeneration, reduced inflammation and pain.
Jo et al. [47]	NCT01300598	RCT, double‐blind, placebo‐controlled (2‐year follow‐up)	20 (high‐dose AD‐MSC *n* = 10; placebo *n* = 10)	Autologous AD‐MSCs	Intra‐articular	100 × 10^6^ (high‐dose)	Saline	WOMAC, KOOS, KSS, VAS, MRI	High‐dose improved pain/function vs. placebo; benefits lasted 2 years
Lamo‐Espinosa et al. [48]	NCT02123368	Multicenter RCT (phase I/II)	30 (BM‐MSC 10M *n* = 10; BM‐MSC 100M *n* = 10; HA *n* = 10)	Autologous BM‐MSCs	Intra‐articular	10 × 10^6^ vs. 100 × 10^6^	HA	WOMAC, VAS, MRI, safety	100M group improved more than HA; safe

### 3.1. Early‐Phase Clinical Studies

Early‐phase clinical studies primarily aim to evaluate safety, feasibility, and preliminarily signals of therapeutic efficacy. Phase I/II work established a favorable safety profile and provided preliminary efficacy signals across autologous and allogeneic approaches. In autologous trials, Jo et al. [10] reported dose‐dependent clinical improvement and MRI cartilage defect filling after AD‐MSC injection, persisting at a 2‐year follow‐up. Lamo‐Espinosa et al. [48] demonstrated superior symptom relief and MRI preservation with BM‐MSCs compared with HA, sustained through 4 years. Shapiro et al. [36] found that bone marrow aspirate concentrate (BMAC) treated showed greater short‐term pain reduction versus contralateral knee saline controls, though structural outcomes were unchanged. Among allogeneic products, Vega et al. [12] demonstrated that BM‐MSCs improved WOMAC pain and function versus HA in a double‐blind RCT, while Kuah et al. [37] evaluated Progenza (allogeneic AD‐MSCs), confirming safety with symptomatic benefit though MRI findings were inconclusive. Collectively, these studies confirmed feasibility and consistent symptomatic improvement, highlighting variability and underpowering for structural endpoints [12, 33–37, 47, 48].

### 3.2. RCTs

RCTs provide the most rigorous framework for evaluating the efficacy and safety of MSC‐based therapies in OA. Autologous AD‐MSCs represent one of the most extensively evaluated cell sources. Jo et al. [47] reported sustained improvements in pain and function with high‐dose AD‐MSCs and partial MRI defect filling over 2 years. In a phase IIb trial, Lee et al. [38] showed that a single IA injection of AD‐MSC significantly improved WOMAC pain, stiffness, and function scores compared with placebo, with MRI evidence of cartilage stabilization and no serious adverse events. Other RCTs and controlled studies of adipose‐lineage products, including adipose progenitors and expanded AD‐MSCs, similarly demonstrated symptomatic benefit with acceptable safety [42, 45].

Autologous BM‐MSCs have shown comparable efficacy signals. In the multicenter RCT by Lamo‐Espinosa et al. [39, 48], both 10 × 10^6^ and 100 × 10^6^ BM‐MSC doses improved VAS and WOMAC at 12 months, with the high‐dose arm sustaining functional benefit and MRI preservation up to 4 years. Prospective studies using two BM‐MSC injections (6.1 × 10^7^ cells total) also reported KOOS improvement and increased cartilage thickness at 12 months with good tolerability [32].

Allogeneic MSC products have advanced in parallel. Vega et al. [12] demonstrated that allogeneic BM‐MSCs outperformed HA in WOMAC improvement, although structural benefits were modest. Matas et al. [40] showed that umbilical cord‐derived MSCs (UC‐MSCs) were safe and effective, with repeated dosing outperforming single injections and MRI suggesting stable or slightly increased cartilage thickness. Allogeneic AD‐MSCs also showed promise. Progenza was well tolerated and associated with symptomatic improvement in a dose‐finding RCT [37], whereas ELIXCYTE achieved significant WOMAC improvement by 24 weeks that persisted to 96 weeks, without major safety concerns, in a multicenter randomized active‐controlled trial [41]. Additional RCTs, including phase IIb and triple‐blind trials, further supported the symptomatic efficacy and safety of adipose‐derived products [43, 46].

Meta‐analyses integrating these trials provide additional evidence for the therapeutic potential of IA MSC injections. Across AD‐MSCs, BM‐MSCs, and UC‐MSCs, treatment significantly improves pain (VAS) and function (WOMAC) compared with HA or placebo, with adverse event rates similar to controls [49, 50]. However, evidence for structural benefits remains less consistent, likely reflecting heterogeneity in the cell source, dose, delivery regimens, and follow‐up duration [12, 33, 34, 36–50].

### 3.3. Key Challenges in Clinical Translation

Barriers to definitive translation remain, as product heterogeneity arising from tissue source, donor variability, and expansion protocols complicates comparisons, and standardized potency assays are still lacking [51]. Optimal dosing and regimen (10^6^–10^8^ cells; single vs. multiple injections) remain unsettled. Most trials are underpowered for structural endpoints, use heterogeneous comparators, and emphasize symptoms over structural modifications. Durability is another concern, as MSCs are rapidly cleared from joints, raising the need for repeat dosing or biomaterial‐assisted delivery. Manufacturing scale‐up, cost, and quality control remain unresolved, and biomarkers or advanced imaging is seldom incorporated despite their potential to clarify mechanisms and stratify responders [12, 32–50].

### 3.4. Lessons for Translation

Several lessons have emerged. First, proof of concept is strong, and IA MSC administration is consistently safe and reproducibly improves symptoms, even when structural modification is inconsistent. Second, immunomodulation appears central, as clinical gains align with suppression of IL‐1β/TNF‐driven inflammation, positioning MSCs as modulators of joint homeostasis rather than direct chondrocyte replacements. Third, standardization is essential across cell source, dosing strategies, potency assays, and outcome assessments [51]. In this context, autologous MSCs offer advantages in immunological compatibility and may support one‐stage procedures, whereas allogenic MSCs enable scalable “off‐the‐shelf” products with more consistent manufacturing. Fourth, durability likely requires innovation (e.g., repeat dosing, hydrogel encapsulation, scaffold‐based delivery, or combination strategies that enhance cell retention and activity). Given the multifactorial nature of OA, MSC therapy alone may not address all aspects of disease pathology and may ultimately need to be integrated with complementary regenerative or disease‐modifying strategies. Finally, embedding biomarkers and advanced imaging into trial design will help define biological activity, enable patient stratification, and support regulatory acceptance.

### 3.5. Synthesis and Outlook

Clinical evidence indicates that MSC therapy safely alleviates OA symptoms, largely through paracrine immunomodulation rather than durable engraftment. This mechanistic understanding helps explain the variability in structural outcomes. Persistent challenges, including limited persistence, manufacturing complexity, and regulatory hurdles, are driving interest in cell‐free modalities such as EVs and exosomes, which may capture MSC paracrine mechanisms in more standardized and scalable formats. These emerging paradigms are the focus of the next section.

## 4. Stem Cell‐Free Derivatives and Exosome‐Based Strategies

### 4.1. Introduction to Stem Cell‐Free Derivatives

Because MSCs act largely through paracrine signaling rather than engraftment, interest has shifted toward stem cell‐free derivatives. These include secretome, purified proteins, extracellular matrix (ECM) scaffolds, and particularly EVs such as exosomes, which encapsulate proteins, lipids, and RNAs that replicate MSC immunomodulatory and chondroprotective effects [52]. Compared with whole‐cell therapies, exosomes offer stability, scalability, and regulatory compatibility [29, 53]. Preclinical studies, summarized in Table 2, confirm their ability to suppress inflammation, protect cartilage, and reduce pain behaviors [30, 31, 54].

**Table 2:** Preclinical trials of MSC‐derived products in OA.

Author	MSC‐derivative type	Cell source	Model	Deliver route	Control	Endpoint	Key findings
Tao et al. [25]	Exosomes (miR‐140‐5p overexpressing)	Human synovial MSCs	Rat OA	Intra‐articular	Unmodified exosomes	Histology, cartilage repair	Engineered exosomes enhanced cartilage regeneration and prevented OA
Cosenza et al. [29]	Exosomes and microparticles	Mouse BM‐MSCs	Mouse OA	Intra‐articular	PBS	Cartilage/bone degradation	EVs protected cartilage and bone from OA damage
Jin et al. [54]	Exosomes	Human BM‐MSCs	Rat OA (surgical DMM)	Intra‐articular injection	PBS	Cartilage integrity, pain, histology	Exosomes reduced cartilage degradation and improved pain
Liu et al. [55]	Exosomes (KLF3‐AS1 enriched)	Human MSCs	Rat OA	Intra‐articular	Vehicle	Cartilage repair, proliferation	Exosomal KLF3‐AS1 promoted cartilage repair and chondrocyte proliferation
Zhang et al. [56]	Exosomes	Human ESC‐MSCs	Rat OA	Intra‐articular	PBS	Proliferation, apoptosis, immune markers	Exosomes enhanced proliferation, reduced apoptosis and modulated immunity
Niada et al. [57]	Secretome (conditioned medium)	Human ADSCs	Human articular chondrocytes (in vitro, TNFα stimulation)	In vitro	TNFα alone	Hypertrophy, catabolic markers	Secretome reduced TNFα‐induced hypertrophy and catabolic gene expression
Wu et al. [58]	Exosomes (miR‐100‐5p enriched)	Human infrapatellar fat pad MSCs	Rat OA	Intra‐articular	Unmodified exosomes	Cartilage protection, gait	Protected cartilage and gait via mTOR inhibition
Zhou et al. [59]	Exosomes	Human BM‐MSC	OA chondrocytes (in vitro)	In vitro	IL‐1β alone	Inflammation, catabolic markers	Exosomes inhibited IL‐1β‐induced inflammation
Mao et al. [60]	Exosomes (miR‐92a‐3p enriched)	Human BM‐MSCs	Mouse OA	Intra‐articular	Unmodified exosomes	Chondrogenesis, WNT5A	Exosomes enhanced chondrogenesis and suppressed degradation
Cavallo et al. [61]	Small EVs (from inflamed MSCs)	Human AD‐MSCs (inflamed)	In vitro OA chondrocytes	In vitro	EVs from normal AD‐MSCs	NF‐κB activation, catabolic markers	Inflamed‐MSC EVs worsened inflammatory/catabolic responses
Wan et al. [62]	Hydrogel‐encapsulated engineered exosomes	Rat BM‐MSCs	Rat OA	Intra‐articular hydrogel	Free exosomes, PBS	Cartilage, pain behavior	Hydrogel improved targeting and therapeutic efficacy
Feng et al. [63]	Modified small EVs (charge‐reversed with εPL‐PEG‐DSPE)	Human iPSC‐MSCs	Mouse OA	Intra‐articular	Native EVs	Cartilage integrity, inflammation	Charge reversal improved targeting and OA efficacy
Yu et al. [64]	Exosomes	Rat BM‐MSCs	Rat OA (tendon defect)	Local fibrin delivery system	Vehicle	Proliferation, apoptosis	Induced TSPCs proliferation, migration and differentiation
Warmink et al. [65]	EVs vs. MSCs	Human BM‐MSCs	Mouse metabolic OA (diet‐induced)	Intra‐articular	MSCs and PBS	Cartilage, synovitis	EVs more effective than MSCs
Hanai et al. [66]	Small EVs (chemically‐defined media)	Human AD‐MSCs	Mouse OA + in vitro human chondrocytes	Intra‐articular	EVs from serum‐containing media	Chondrocyte viability, cartilage	Defined‐media EVs showed stronger bioactivity and OA benefit

### 4.2. Preclinical Evidence for EVs in OA

Extensive studies demonstrate that MSC‐derived exosomes reproduce many benefits of whole‐cell therapy in OA models. BM‐MSC exosomes reduce cartilage degradation, suppress synovitis, and restore anabolic markers such as COL2A1 while downregulating MMP‐13 and ADAMTS‐5 [29, 54]. Exosomes from adipose, synovial, and infrapatellar fat pad MSCs similarly enhance chondrocyte proliferation, reduce apoptosis, and modulate inflammation [25, 55–59].

Engineering further enhances potency; synovial MSC exosomes enriched with miR‐140‐5p promoted cartilage matrix synthesis [25]; BM‐MSC exosomes carrying miR‐92a‐3p suppressed WNT5A signaling and catabolism [60]; and other modifications such as KLF3‐AS1 [55], miR‐100‐5p [58], or miR‐455 [67] improved chondroprotection. In large‐animal models, Wharton’s jelly MSC exosomes combined with ECM scaffolds enhanced osteochondral repair and polarized macrophages toward an anti‐inflammatory phenotype [68]. Notably, EVs from inflamed adipose MSCs amplified NF‐κB activation and catabolism, highlighting the importance of source and manufacturing conditions (63)

### 4.3. Early Clinical Studies of EVs in OA

Human evidence remains limited. A pilot study of ExoFlo (BM‐MSC–derived exosomes) reported improved pain and function at 6 months with no serious adverse events [69]. Broader surveys show that over 40 EV‐related clinical studies have been initiated, most remain early phase, and none have advanced to late‐stage OA testing [53]. These findings establish feasibility and safety but leave uncertainties in dosing, regimen, and biomarkers.

### 4.4. Delivery Strategies for EVs

A key barrier is the rapid clearance of EVs from the joint. Bioengineering approaches have been developed to prolong retention and enhance the efficacy. Hydrogels such as GelMA sustain exosome activity and attenuate OA severity in vivo [62, 70]. Surface charge modification enables deeper cartilage penetration and more efficient delivery [63, 71]. Scaffold integration localizes vesicles within defect sites, coupling structural support with biological signaling [68, 72]. Emerging smart carriers, including magnetic and stimuli‐responsive systems, further improve targeting and adaptive release [63, 73].

### 4.5. Translational Implications

MSC‐derived exosomes have emerged as the leading stem cell‐free candidates for OA, combining safety, scalability, and mechanistic potency [52, 69]. Successful translation will depend on rigorous standardization guided by frameworks such as MISEV 2023 [74], optimized delivery platforms [62, 63, 71, 73], and incorporation of biomarkers to monitor biological activity. Combining exosomes with scaffolds, hydrogels, or genetic engineering offers the potential for durable disease modification [67, 68, 75].

### 4.6. Summary and Outlook

MSC‐derived EVs have emerged as a promising cell‐free approach that may recapitulate some of the paracrine mechanisms of MSC therapy while offering potential advantages in safety and manufacturability. However, EV‐based therapies also face important challenges, including variability in EV composition depending on the cell source and culture conditions, incomplete characterization of bioactive components, and the lack of certain functions associated with living cells. Further work is therefore required to better define EV potency, optimize production and delivery strategies, and determine their therapeutic role relative to MSC‐based therapies in OA.

## 5. Bioengineering Platforms and Innovations

**Figure 2:**
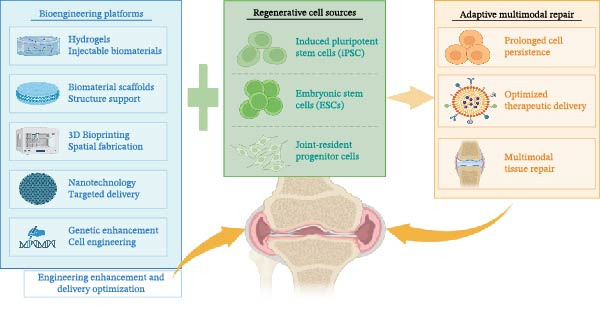
Bioengineering strategies and stem cell sources enabling multimodal joint regeneration. Created with BioRender.com.

Both MSC‐based and EV‐based therapies face key translational barriers, such as short IA persistence, heterogeneous potency, and limited structural benefit. These challenges have driven bioengineering innovations aimed at prolonging therapeutic effects, enhancing targeting, and integrating regenerative cues into rationally designed delivery systems. Advances in hydrogels, scaffolds, genetic engineering, 3D bioprinting, and nanotechnology are converging to transform empirical injections into more precise and durable approaches for OA (Figure 2). Representative preclinical studies of these platforms are summarized in Table 3, underscoring their potential to overcome current limitations and accelerate clinical translation.

**Table 3:** Preclinical bioengineering innovations for MSC‐ and EV‐based therapies in OA.

Author(s)	Innovation and source	Description and key mechanism	Key findings
Jiang et al. [68]	Bioactive nanoparticles	Injectable nanoplatform loaded with MSC‐derived exosomes	Modulates inflammation and enhances cartilage matrix synthesis in OA models
Zhang et al. [76]	miRNA‐enriched exosomes	Exosomes enriched with miR‐140 for cartilage regeneration	Promoted cartilage repair and regeneration in OA animal models
Zhou et al. [77]	Exosome mechanism study	Investigated MSC exosome‐mediated cartilage repair via CD73‐mediated AKT/ERK activation and M2 macrophage polarization	Enhanced proliferation, matrix synthesis, and anti‐inflammatory response in osteochondral defects
Rey‐Rico et al. [78]	Graphene‐based scaffold	Graphene scaffold supporting MSC osteogenic differentiation	Accelerates osteogenesis without impairing proliferation
Wagenbrenner et al. [79]	Injectible biomaterial gel	Mesoporous bioactive glass/PCL scaffold	Promotes bone regeneration in osteoporotic sheep in vivo
Clark et al. [80]	Integrin‐specific hydrogels	Synthetic hydrogel with integrin‐targeting peptides	Enhances MSC survival, paracrine signaling, and osteoreparative activity
Gilroy et al. [81]	Stimulus‐responsive scaffolds	Scaffolds responsive to mechanical load or magnetic fields	Dynamically activates MSCs via rehabilitation synergy
Han et al. [82]	Multifunctional bioactive hydrogel	Hydrogel with immunomodulatory and chondrogenic cues	Promoted MSC chondrogenesis, reduced OA inflammation, and improved cartilage repair
Li et al. [83]	Engineered EVs	EVs modified for cartilage targeting and enhanced cargo delivery	Protected cartilage and reduced OA progression in vivo
Yang et al. [84]	Nanoengineered EVs	EVs with targeting peptides and nanocarriers	Enhanced cartilage uptake and disease‐modifying efficacy in OA
Zhang et al. [85]	IL 4 loaded hydrogel scaffold	Hydrogel delivering IL 4 to reprogram macrophages to an M2 phenotype	Reduces inflammation and promotes MSC‐mediated cartilage repair
Pang et al. [86]	GelMA hydrogel + MSC exosomes	GelMA hydrogel sustaining exosome release	Reduced OA severity in vivo, enhanced matrix synthesis, suppressed catabolic factors, and induced M2 polarization
Cao et al. [87]	Exosome‐hydrogel nanoplatform	Engineered exosome‐loaded injectable hydrogel	Provided sustained release; improved cartilage repair and reduced inflammation in vivo

### 5.1. Hydrogels for Sustained Delivery

Rapid clearance limits the efficacy of MSCs and EVs. Hydrogels provide biocompatible matrices that encapsulate cells or vesicles, preserve bioactivity, and enable controlled release. Both natural polymers (e.g., HA, alginate, and gelatin) and synthetic variants (PEG and GelMA) can be tailored for joint applications [64]. Hydrogel‐exosome systems prolong local activity and enhance cartilage and bone repair [88–90]. Examples include adhesive hydrogels that recruited endogenous cells to promote cartilage repair [76] and thermosensitive hydrogels releasing TGF‐β1 supported cartilage regeneration [77]. On the whole, hydrogels extend IA residence while enabling the combinatorial delivery of MSCs, EVs, and bioactive molecules [78, 79].

### 5.2. Scaffold Integration for Structural and Biological Repair

Scaffolds complement hydrogels by providing structural integrity alongside biological delivery. Constructed from ECM, synthetic polymers, or composites, scaffolds guide tissue regeneration and integrate with cartilage and subchondral bone. When functionalized with MSCs or EVs, they combine mechanical and biological repair [80, 81]. In preclinical models, exosome‐functionalized ECM scaffolds improved osteochondral regeneration while reducing synovial inflammation [68]. Decellularized ECM scaffolds carrying EVs activated reparative signaling pathways such as 4E‐BP [82], while 3D‐printed, exosome‐enriched constructs achieved robust osteochondral regeneration [83]. These studies underscore scaffolds as versatile platforms for durable, integrative regeneration [91].

### 5.3. Cargo Engineering and Genetic Enhancement

Engineering strategies can enhance the therapeutic potency of MSCs and EVs. MicroRNA enrichment is particularly effective; for example, exosomes overexpressing miR‐140‐5p promoted ECM synthesis and delayed OA progression [25]; miR‐92a‐3p suppressed catabolic enzymes and improved cartilage histology [60]; and miR‐455 augmented anabolic signaling in vivo [67]. Beyond EVs, MSCs can be genetically modified to overexpress cytokines (IL‐1Ra, IL‐10, and stkIL‐1R2) or transcription factors (SOX9), generating “designer cells” with tailored paracrine outputs [84, 85, 92]. Such modifications shift MSC therapy from symptomatic relief toward true disease‐modifying potential.

### 5.4. 3D Bioprinting and Tissue Engineering

Given the complexity of OA pathology, which involves both cartilage and subchondral bone, 3D bioprinting offers a means to replicate the osteochondral architecture. This technology enables spatially precise placement of cells, EVs, and biomaterials to mimic native joint layering [91, 93, 94]. Bioprinted, multilayered constructs promoted chondrogenesis and mineralization [95]. In vivo, GelMA‐MSC scaffolds repaired rabbit cartilage defects with improved integration [96], while GelMA hydrogels carrying MSC‐derived nanovesicles reduced OA severity by promoting chondrogenesis and inducing M2 macrophage polarization [86]. Bioprinting thus merges structural precision with biological activity, enabling personalized regenerative therapies.

### 5.5. Nanotechnology and Smart Systems

Nanotechnology further refines MSC/EV delivery by enabling deeper tissue penetration and stimulus‐responsive release. Charge‐reversal exosomes penetrated dense cartilage and formed intratissue depots [71]. Stimuli‐responsive carriers released therapeutic cargo in response to pH, ROS, or enzymatic activity, maximizing local efficacy while minimizing systemic exposure [97, 98]. Bio‐nanoparticles loaded with synovial MSC exosomes mitigated oxidative stress and slowed OA progression [87], while magnetic polysaccharide carriers improved exosome retention and synergy [84]. These “smart” systems align with precision medicine, providing adaptive and targeted delivery.

### 5.6. Clinical Translation Pathways

The convergence of hydrogels, scaffolds, genetic engineering, 3D bioprinting, and nanotechnology marks a transition from empirical MSC injections to rationally engineered regenerative systems [79]. Overall, these platforms address key limitations observed in clinical trials, including short persistence, inconsistent structural benefit, and variable potency. Early feasibility studies in large‐animal models have confirmed the safety and efficacy of engineered scaffolds, bioactive hydrogels, and exosome‐functionalized biomaterials [86]. Successful clinical translation will require harmonized manufacturing standards, regulatory approval, and incorporation of biomarker. Ultimately, coupling cell‐based therapies with advanced biomaterials and smart delivery systems may enable adaptive, personalized, and truly disease‐modifying OA treatments.

## 6. Beyond MSCs: Emerging Stem Cell Sources for OA Therapy

Advances in delivery platforms, scaffolds, and nanotechnologies have refined MSC‐based therapies, yet questions remain as to whether MSCs are the optimal cell source for durable disease modification. This has stimulated interest in alternative stem cell populations, including induced pluripotent stem cells (iPSCs), embryonic stem cells (ESCs), and tissue‐specific progenitors, that may offer enhanced regenerative potential, more consistent manufacturing, or tailored safety advantages.

### 6.1. iPSCs

iPSCs provide an inexhaustible, customized cell source capable of differentiating into cartilage‐ and bone‐relevant lineages. Preclinical studies show promise, human iPSC‐derived cartilage particles integrated into rodent and mini‐pig defects without tumorigenesis [99], while allogeneic iPSC‐derived organoids were engrafted into primate joints with immune tolerance over 4 months [100]. iPSC‐derived chondrocytes also exhibit juvenile‐like features, including enhanced proliferation and resistance to IL‐1β [101]. Translational barriers remain, particularly tumorigenicity, genomic instability, and complex manufacturing, but initiatives such as HLA‐matched iPSC banks may enable standardized, “off‐the‐shelf” therapies.

In addition to cell‐based approaches, EVs derived from iPSC have also attracted attention as potential cell‐free therapeutics. Preclinical studies suggest that iPSC‐derived EVs can promote chondrocyte proliferation, suppress inflammatory signaling, and enhance cartilage matrix synthesis in experimental OA models [102], highlighting their potential as an alternative regenerative strategy.

### 6.2. ESCs

ESCs share the pluripotency and chondrogenic capacity of iPSCs, with defined, feeder‐free protocols producing potent chondroprogenitors [103]. Reviews emphasize their ability to recapitulate developmental chondrogenesis [104]. Nonetheless, ethical concerns, tumor risk, and differentiation variability have restricted their use to preclinical studies. ESCs are likely to remain most valuable as research tools for modeling cartilage development and OA pathogenesis rather than near‐term clinical therapies.

Similar to MSCs and iPSCs, ESC‐derived EVs are also being explored as potential cell‐free therapeutics. Early preclinical studies indicate that ESC‐derived EVs can modulate inflammatory pathways and support cartilage repair processes [105], although their therapeutic potential in OA remains largely at an exploratory stage.

### 6.3. Tissue‐Specific Progenitors

Joint‐resident progenitors may offer developmentally primed options for cartilage repair. Synovial‐derived stem cells (SDCs) demonstrate greater chondrogenic potential than BM‐ or AD‐MSCs, producing larger pellets rich in type II collagen [106]. Cartilage‐resident progenitor cells (CRPCs) migrate to injury sites and support endogenous repair, although their function declines in the inflammatory OA milieu [107]. Clinical use is limited by scarcity and invasive harvesting, but in vitro expansion and scaffold‐based delivery approaches are under exploration.

### 6.4. Challenges and Opportunities

Emerging stem cell populations broaden the regenerative toolkit for OA. iPSCs offer renewable, customizable platforms but require stringent safeguards to ensure safety and manufacturing consistency. ESCs possess robust chondrogenic potency yet face persistent ethical and immunological barriers. For both pluripotent stem cell‐derived therapies, additional safety concerns include the risk of tumorigenicity and the need for precise control of differentiation. Tissue‐specific progenitors are developmentally primed for joint repair but remain difficult to source and may be vulnerable in the diseased OA environment. In addition to cell‐based approaches, EVs derived from these stem cell populations may provide attractive cell‐free therapeutic options. However, their clinical translation faces additional challenges, including variability in EV composition, incomplete understanding of active components, and the lack of standardized production and characterization protocols.

Future progress will likely depend on integration with bioengineering platforms, such as hydrogels and scaffolds, to improve the survival and enhance therapeutic function. These emerging stem cell sources may complement current MSC‐based strategies, although further studies are needed to clarify their safety, manufacture feasibility, scalability, and therapeutic efficacy in OA models and clinical settings. Whether these approaches will ultimately outperform MSC‐based therapies remains uncertain, as their differentiation potential must be balanced against challenges in safety, manufacturing complexity, and regulatory acceptance.

## 7. Future Directions and Integrative Perspectives

Although MSCs remain the most extensively studied cell type for OA therapy, other regenerative cell sources are increasingly being investigated. iPSCs and ESCs offer pluripotent differentiation potential, whereas tissue‐specific progenitors may provide developmentally primed repair capacity. However, each approach presents distinct limitations related to safety, scalability, or sourcing. Future progress will likely depend on integrating optimized cell sources with improved delivery strategies, biomarker‐guided patient stratification, and regulatory pathways that enable rigorous clinical translation [6, 52].

### 7.1. From Symptom Relief to Disease Modification

To date, most stem cell‐based interventions for OA have primarily achieved symptomatic relief, including reducing pain, improving function, and enhancing quality of life, while consistent structural modification of cartilage and subchondral bone remains elusive. Early MSC trials confirmed safety and feasibility but showed variable regenerative impact, likely limited by rapid clearance and heterogeneous potency [47, 48]. Similarly, EV‐based therapies suppress inflammation and pain but have demonstrated only modest tissue repair in vivo [29, 54].

Achieving true disease modification, including sustained structural regeneration and slowed disease progression, will likely require improving the persistence of therapeutic signals within the joint, either through optimized dosing strategies or delivery systems that prolong MSC or EV activity in the OA microenvironment. In addition, biologically informed patient selection may be critical, as OA is a heterogeneous disease with substantial variation in inflammatory activity, structural damage, and progression patterns among patients. Biomarker‐guided stratification based on inflammatory phenotype, disease stage, and molecular profiles may therefore help identify patient subgroups most likely to respond to MSC‐ or EV‐based therapies and improve the likelihood of achieving durable disease modification.

### 7.2. Integrating Cell, Cell‐Free, and Bioengineering Strategies

No single modality, such as MSCs, EVs, or progenitors, has yet achieved durable outcomes in OA. Evidence suggests that integration, rather than isolation, will drive progress. Whole‐cell therapies provide a broad paracrine repertoire, EVs capture immunomodulatory signals in a safer, standardized form, and bioengineering strategies extend persistence, enhance targeting, and support tissue architecture [66, 70]. Preclinical studies demonstrate that multimodal techniques, such as EV‐loaded hydrogels or progenitor‐seeded scaffolds, outperform single platforms [68, 86]. Future therapies will likely combine these strengths to achieve adaptive and durable repair.

### 7.3. Biomarkers and Patient Stratification

A major barrier is the lack of reliable biomarkers to identify responders and monitor biological activity. Most trials still rely on symptoms, while imaging is insensitive to early changes. Candidate molecular markers, such as lipocalin‐2 (LCN2/NGAL), cartilage breakdown products, such as CTX‐II and COMP, and inflammatory mediators, show promise [92, 108]. Quantitative MRI methods for cartilage composition provide complementary, noninvasive readouts [109]. Incorporating biomarkers into the trial design will enable patient stratification, clarify mechanisms, and align endpoints with regulatory standards.

### 7.4. Translational and Regulatory Challenges

Despite progress, translation faces regulatory and logistical hurdles. For MSCs, donor variability, expansion protocols, and absent potency assays complicate standardization [51]. EVs align more closely with biologics frameworks but lack consensus on manufacturing and release criteria [74]. Safety concerns persist, including tumorigenicity in pluripotent products and hypertrophy in progenitors, necessitating long‐term monitoring. Regulatory bodies still prioritize symptomatic outcomes, relegating structural endpoints to exploratory status (US FDA, 2018). Cost and scalability further limit access: autologous therapies are resource‐intensive, whereas allogeneic products raise immunologic concerns [110]. Addressing these issues will require harmonized manufacturing standards, adaptive trial designs, and early engagement with regulators.

### 7.5. Integrative Perspectives

In summary, these considerations suggest that the future of stem cell therapy for OA will depend on convergence rather than a single breakthrough. MSCs, EVs, pluripotent cells, and joint‐specific progenitors each contribute distinct strengths, while biomaterials extend their persistence and precision. The field is moving toward rationally engineered regenerative systems, guided by biomarkers and aligned with regulatory frameworks. The most promising strategies will likely be combinative and adaptive, integrating cell or cell‐free products with hydrogels, scaffolds, and smart delivery systems while tailoring treatment to patient subgroups through precision diagnostics. Such integrative approaches hold the potential not only to relieve symptoms but also to promote more durable, disease‐modifying repair in OA [67, 72].

## 8. Conclusion

Stem cell‐based therapies are redefining OA treatment, shifting the field from symptomatic relief toward regenerative and potentially disease‐modifying strategies. Among these, MSCs remain the most clinically advanced, yet the rise of cell‐free derivatives and bioengineered delivery systems points toward safer, more scalable, and durable solutions. Emerging sources such as iPSCs, ESCs, and joint‐specific progenitors further broaden the regenerative landscape, offering complementary opportunities. Ultimately, success will depend on integrating these advances within standardized manufacturing frameworks, guided by biomarkers and supported by regulatory alignment. Such convergence promises to deliver personalized and durable regenerative therapies capable of fundamentally altering the trajectory of OA care.

## Funding

No funding was received for this manuscript.

## Conflicts of Interest

The authors declare no conflicts of interest.

## Data Availability

Research data are not shared.
